# Effects of Aerobic Training on Brain Plasticity in Patients with Mild Cognitive Impairment: A Systematic Review of Randomized Controlled Trials

**DOI:** 10.3390/brainsci12060732

**Published:** 2022-06-02

**Authors:** Farid Farhani, Shahnaz Shahrbanian, Mohammad Auais, Amir Hossein Ahmadi Hekmatikar, Katsuhiko Suzuki

**Affiliations:** 1 Department of Sport Science, Faculty of Humanities, Tarbiat Modares University, Tehran P.O. Box 14115-111, Iran; f.farid1993@yahoo.com (F.F.); a.ahmadihekmatik@modares.ac.ir (A.H.A.H.); 2 Faculty of Health Sciences, School of Rehabilitation Therapy, Queen’s University, Kingston, ON K7L 3N6, Canada; mohammad.auais@queensu.ca; 3 Faculty of Sport Sciences, Waseda University, Tokorozawa 359-1192, Japan

**Keywords:** aerobic training, cognitive impairment, neurotrophic factors, hippocampal, brain plasticity

## Abstract

The purpose of this study was to systematically review to find if aerobic exercise compared to no exercise or any other intervention affects brain plasticity among people with mild cognitive impairment (MCI). Searches were conducted in the Scopus, SciELO, PubMed, Web of Science, Science Direct, and Google Scholar databases. The included studies were randomized control trials (RCTs) written in English comprising individuals with MCI that evaluated the effects of aerobic training on brain-derived neurotrophic factor (BDNF), brain structures, or brain activity. The quality of trials was evaluated using the PEDro scale for RCTs. Twelve studies with medium to high quality were included, of which five studies focused on brain-derived neurotrophic factor (four articles reported elevation and one article reported no changes in BDNF levels following the aerobic exercise), two studies focused on brain structures (both reported increases in hippocampus volume following the aerobic exercise), and five studies focused on brain activity (four articles reported positive changes, and one article reported no changes in brain activity following the aerobic exercise). Research regarding the effects of aerobic training on brain plasticity in people with MCI is in its infancy. Still, aerobic exercise seems to be a promising therapy in people with MCI.

## 1. Introduction

Mild cognitive impairment (MCI) is a heterogeneous clinical syndrome that reflects changes in cognitive function and defects in neuropsychological tests. MCI is more likely to occur at any age and is more common in the elderly [1]. Numerous international studies estimated the annual prevalence of MCI to range from 12% to 18% in persons older than 60 years [2,3].

The initial definition of MCI directly concerned the detection of underlying Alzheimer’s disease (AD) [4]. Patients with MCI present with changes in brain plasticity (e.g., neurochemical, neuroplasticity, and neurostructural modifications), which is a continuous alteration of the neural pathways and synapses of the living brain and nervous system in response to injury, many times before the onset of plain AD [5].

The hippocampus, which is critical for acquiring new memories and learning, is affected by aging and age-related MCI [6]. At age 85, the risk of developing MCI during life without the APOE phenotype (an essential protein in the hippocampus) is 11% in men and 14% in women [7]. Brain plasticity allows the central nervous system to acquire additional information and learn skills, reorganize neuronal networks in response to environmental stimulation, and recover from brain injuries [8]. The primary and essential mechanisms involved in plasticity include neurochemical and electrophysiological manifestations (e.g., neurogenesis, programmed cell death, neurotransmitter release, and long-term potentiation (LTP)). In general, these changes are adaptive and beneficial but can also trigger negative alterations in the brain in some situations [9]. MCI is a syndrome that may be caused by many underlying causes, one of which is AD. No disease-modifying treatment for MCI exists yet, but efforts within the last decades have aimed at developing pharmacological therapies targeted at reducing cortical amyloid-þ (Aþ) and tau pathology, which are believed to be key in the pathogenesis of AD [10]. There is growing evidence that non-pharmacological interventions help in preserving personal autonomy, reducing neuropsychiatric symptoms, and improving the quality of life in MCI patients [11,12,13]. Moreover, the evolving literature has shown significant benefits of physical exercise, attenuating cognitive impairment, reducing dementia progression, and increasing brain-derived neurotrophic factor (BDNF) and hippocampal plasticity [14,15]. Exercise has been shown to increase the expression of BDNF, which may stimulate hippocampal plasticity [15].

Many experimental studies showed potential mechanisms to account for cognition improvement due to exercise, including effects on neuronal survivability and function, neuroinflammation, vascularization, the neuroendocrine response to stress and brain amyloid burden, and physiological processes, such as glucoregulation and the cardiovascular system [16]. Brain imaging studies suggested that aerobic fitness in healthy elderly individuals is associated with reduced age-related atrophy and increased perfusion in brain areas sustaining executive functions and memory, resulting in less vulnerability to the effects of aging [17,18]. Previous meta-analyses of randomized control trials (RCTs) showed that physical exercise in healthy adults is associated with cognitive improvement, larger hippocampal volumes, an attenuation of age-related grey matter volume loss, and an improved connectivity of brain networks [19].

To date, controversy remains regarding the benefits and mechanisms underpinning the effects of aerobic exercise on patients with MCI. Hence, differences regarding speed, the duration of the training, and acute versus chronic training are potential sources of divergent conclusions [20,21]. Nevertheless, the neurophysiological effects of aerobic exercise, conditions of health, and pathology of the brain should be distinctly investigated. Experimentation could provide the unique opportunity to examine brain plasticity alterations after aerobic exercise.

Current studies commonly investigate brain plasticity, which is associated or not with behavior in normal conditions of health and in MCI. This is understandable given the legitimate concern regarding brain alterations associated with aging and MCI. This review focuses on the brain areas that exhibit extensive functional plasticity in adaptation to aerobic exercise. The objective of this review is to summarize the current evidence regarding the effects of aerobic exercise on brain plasticity. The PICO format of the question was: among people with MCI (P), does aerobic exercise (I) in comparison to no exercise or any other kind of intervention (C) affect brain plasticity (O)?

## 2. Materials and Methods

This systematic review was conducted in accordance with the preferred reporting items for systematic review (PRISMA) guidelines.

### 2.1. Eligibility Criteria

Randomized controlled trials in patients with MCI that compared the effects of aerobic exercise on brain plasticity with no invention or any other intervention were included. Brain plasticity was defined as the capacity to continuously alter the neural detected pathways, which included brain-derived neurotrophic factor, brain structure changes, and brain activity. The search was limited to original articles published in the English language. We did not consider grey literature, e.g., abstracts from conferences, government documents, and reports. The search was not limited by the aerobic exercise training type, duration, frequency, intensity, or time to ensure we did not miss any relevant articles.

### 2.2. Data Sources and Search Strategy

Comprehensive and systematic searches were conducted by two independent authors (F.F. and Sh.Sh.) using the following electronic databases: MEDLINE (via PubMed), SCIENCE DIRECT (Web of Science), Pedro, Cochrane Library, and SCOPUS as well as the Google Scholar search engine. The following MESH terms and search terms were used: [hippocamp] OR [brain] AND [alzheimer] OR [dementia] OR [mild cognitive impairment] AND [treadmill] OR [aerobic exercise] OR [aerobic] OR [physical exercise] OR [exercise training] AND [randomized clinical trials]. Electronic databases were searched from inception to 27 April 2022. Manual searches of the reference lists from the included articles were also conducted.

### 2.3. Data Extraction

Two authors (F.F. and Sh.Sh.) independently screened the title and abstract of every citation found in the literature search. At first, the titles and abstracts clearly dealing with a different subject were excluded. All other data were extracted directly from the full-text articles, and those with potential relevance were examined for eligibility criteria. Disagreements were resolved by consensus. Crude agreement and Cohen’s kappa coefficient were used to assess the inter-rater agreement between the two reviewers at each major step of the review from study selection to quality assessment [22].

### 2.4. Quality Assessment

The PEDro scale for RCT articles was used to evaluate the quality of the articles [23]. The Physiotherapy Evidence Database (PEDro) scale includes the 11 following criteria: specification of eligibility criteria for subjects; random and concealed allocation of subjects to groups; baseline similarity of the groups regarding the most important prognostic indicators; blinding of subjects, therapists, and assessors; measuring at least one key outcome from more than 85% of the subjects initially allocated to groups; reporting the results of between-group statistical comparisons for at least one key outcome; and providing both point estimates and measures of variability for at least one key outcome. The quality of each article was rated from 0 to 10. The studies in this method with a scoring of 6 to 10 were considered methodologically “high,” 4 to 5 were considered “fair,” and ≤3 were considered “poor” [24]. Moreover, the level of evidence was determined using the method by Sackett et al. [25]. Level 1a of evidence, or strong, was given if two or more “high” quality RCTs based on the PEDro scale (PEDro ≥ 6) showed the positive effect of the specific intervention on a study outcome. Level 1b, or moderate, was given when one RCT of “high” quality existed based on the PEDro scale (PEDro ≥ 6), 2a (limited) was given when at least one “fair” quality RCT existed (PEDro = 4–5), and 2b (limited) was given when at least one “poor” quality RCT (PEDro b4) indicated an intervention could be effective [25].

## 3. Results

### 3.1. Number of Papers Sourced and Descriptive Findings

**Figure 1:**
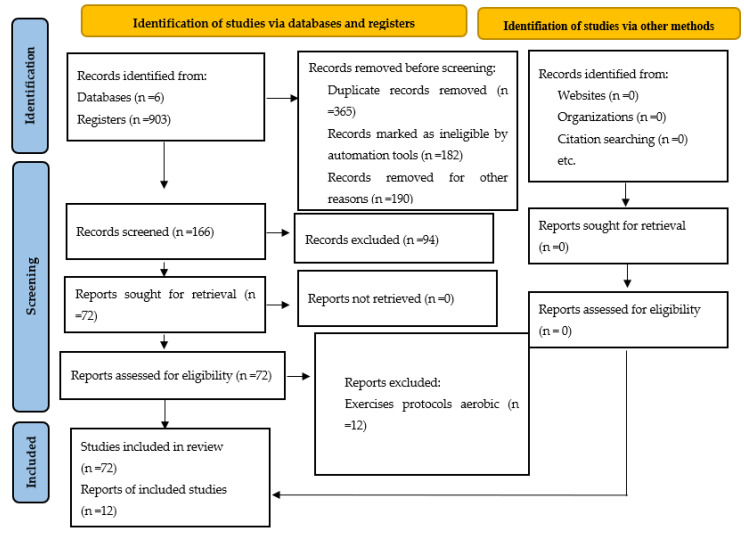
Diagram flow of outcomes of review.

The search strategy retrieved 903 records, and 365 studies were omitted because they were duplicates. Then, out of the remaining 538 studies, 372 studies were excluded after screening and title and/or abstract analysis. Then, out of the remaining 166 studies, 18 studies were excluded for the following reasons: (i) 52 studies had no full-text copies available and (ii) 42 studies were not published in English. At the end of the process, 12 publications meeting the eligibility criteria were included for analysis. Figure 1 depicts the diagram flow of outcomes of the review.

Five studies focused on the effect of aerobic training (four studies of running [12,17,26,27] and one study on walking [11]) on BDNF; two studies focused on the effect of running on hippocampus volume [28,29], and five studies included the effect of aerobic training (aerobic dance [13,30], Baduanjin exercise [31], and running [32,33]) on brain activity (e.g., event-related potential). The characteristics of the 12 studies regarding clinical characteristics, aerobic training protocol, and results are reported in Table 1. There were 447 participants included in the reviewed studies. Regarding gender, 277 (62%) participants were female. Two studies included only females [11,17], whereas one included only males [27]. The participant’s ages were between 55 and 80 years. MCI diagnosis in two studies [17,31] was based on the Peterson diagnostic criteria for MCI [3], whereas 10 studies [4,11,12,13,26,28,30,32,33] used the neuropsychological test battery and clinical criteria for MCI [34]. Mini-Mental State Exam (MMSE) scores of MCI subjects were reported in only eight studies (between 21 and 30, see Table 1) [11,12,28,29,30,32,33], and Montreal Cognitive Assessment (MoCA) scores were reported in only five studies (between 19 and 25, see Table 1) [13,29,30,31,32]. The control groups were usual care only in six studies [4,11,13,29,30,32], stretch training in five studies [12,17,26,28,33], and usual physical activity in one study [31]. In studies that reported BDNF levels [4,11,17,26], serum BDNF levels were quantitatively determined using the human BDNF ELISA kit (see Table 2). In studies that reported structures of the brain [28,29], the hippocampus volume was quantitatively determined using magnetic resonance imaging (MRI) (see Table 3 and Table 4).

The reviewed studies used running (eight studies), dance (two studies), walking (one study), and Baduanjin (one study) training (see Table 1); the two studies for running [12,26] had the same exercise training protocol, and two studies for aerobic dance [13,30] had the same exercise training protocol. The frequency of exercise sessions was three or four sessions per week in the studies. In most studies, the intensity of exercise was moderate to high and was designed based on the maximum heart rate, heart rate reserve, and maximum oxygen consumption. The overload of exercise intensity was reported in five studies [11,12,17,26,29] and was considered constant in them. The duration of the training period ranged between 2 and 12 months. Moreover, the time of exercise was considered constant in most studies, as six of them reported that each training session lasted for 60 minutes or less (see Table 1) [8,11,12,17,26,32].

### 3.2. Methodological Quality

The PEDro scale for the included studies ranged between 4 and 7 (Table 5).

### 3.3. Classification of Evidence

#### 3.3.1. Brain-Derived Neurotrophic Factor

Four studies with fair [4,12] to high quality [17,26] support that aerobic training has a significant effect on increasing the level of BDNF. Thus, the level of 1a evidence is given to the positive effect of aerobic training on BDNF in patients with MCI.

#### 3.3.2. Brain Structures

Two studies with high quality [28,29] support that aerobic training has a significant effect on increasing the hippocampus volume. Thus, the level of 1a evidence is given to the positive effect of aerobic training on brain structures in patients with MCI.

#### 3.3.3. Brain Activity

Five studies with fair [30,32] to high quality [13,31,33] support that aerobic training has a significant effect on increasing brain activity. Thus, the level of 1a evidence is given to the positive effect of aerobic training on brain activity in patients with MCI.

## 4. Discussion

This study summarized the results of RCTs that examined the effect of aerobic training on the pathophysiology of MCI with an emphasis on brain plasticity. The final search found 12 studies on the effects of exercise training on brain-derived neurotrophic factor, brain structures, and brain activity profiles in persons with MCI. Overall, most studies support a substantial impact of aerobic training on an upregulation of brain plasticity in persons with MCI.

Complex biochemical cascades are responsible for building new vascular and neural structures in the brain. A detailed discussion of these waterfalls is beyond the scope of this study [35]. However, one of the major well-established growth factors is BDNF, which can be discussed in this brief study [36]. BDNF plays an essential role in maintaining synaptic plasticity in learning and memory. BDNF plays a vital role in facilitating nerve growth and maturation through the developmental stages and the regulation of synaptic transmission and flexibility in adulthood [37,38]. BDNF is mainly synthesized in neurons and glial cells and is then transported to presynaptic terminals and postsynaptic dendrites in the brain. The localization of BDNF and its receptor, tropomyosin receptor kinase B (TrkB), to glutamate synapses regulates neurotransmitter release, ion channel activity, axonal pathfinding, and neuronal excitability [39]. It is clear that BDNF levels change in patients with Alzheimer’s disease (AD) [39,40]. BDNF serum concentration is reported to be significantly reduced in patients with severe dementia compared to control subjects [41]. BDNF mRNA is distributed throughout the central nervous system (CNS), including areas of the cortex, hippocampus, substantia nigra, amygdala, and thalamus [42,43]. The pathways associated with changes in neuronal excitability are triggered by the binding of mBDNF to TrkB, indicating that TrkB activation is crucial for controlling the survival, morphogenesis, and plasticity of neurons [44]. In addition, BDNF/TrkB generates many downstream intracellular signaling pathways, such as mitogen-activated protein kinase/extracellular signal-regulated protein kinase (MAPK/ERK), PI3K, and phospholipase Cγ/protein kinase C (PLCγ/PKC) These signaling pathways are associated with the activation of the CREB transcription factor, which mediates the transcription of genes essential for synaptic plasticity [44]. Under pathological conditions such as AD, BDNF is involved in Aβ accumulation, tau phosphorylation, the neuroinflammatory response, and apoptosis [37]. In particular, Aβ has been shown to disrupt BDNF processing both activity-dependently and activity-independently [37]. Recent studies have shown that there is a positive relationship between PA and the concentration of neurotrophic factor in different parts of the body, such as the brain, blood, and muscles [45,46,47,48]. Among the numerous well-known exercise training interventions developed throughout these past years, there seems to be a body of evidence reporting that aerobic exercise appears to increase the expression of NTs, particularly BDNF [49]. One meta-analysis indicated that physical exercise can be a therapeutic choice to upregulate BDNF in patients with MCI and AD. However, in this article, three studies showed a high risk of attrition bias and two showed a high risk of reporting bias [50].

Among the reviewed studies (Table 2), five studies investigated the effect of aerobic training on BDNF in patients with MCI. Four studies with moderate to high quality reported a significant increase in BDNF, and one high-quality study reported no change. Fungwe et al. investigated the effects of six months of running on BDNF of patients with MCI at a frequency of three days per week and a training intensity of 50 to 70% of the VO_2max_ of overload with a training time of 20 to 40 minutes of overload, which showed a significant increase in BDNF [12]. Prior to this study, Baker et al. [17] (training protocol; intervention duration: 6 months, sessions per week: 4, intensity of training: 40 to 80% of HRmax of overload, time of training: 45 to 60 min of overload), Allard et al. [26] (training protocol; intervention duration: 6 months, sessions per week: 3, intensity of training: 50 to 70% of VO_2max_ of overload, time of training: 20 to 40 min of overload), and Kohanpour et al. [27] (training protocol; intervention duration: 12 weeks, sessions per week: 3, intensity of training: 75 to 85% of HRmax, time of training: 8 to 26 min of overload) showed significant increases in the BDNF in MCI patients were confirmed by running. On the other hand, the study of Damirchi et al. [11] investigated the effect of walking on the BDNF of patients with MS. After three weeks of walking with a training intensity of 55 to 75% of the HRmax of overload with a training time of 6 to 20 min of overload for three days per week, they did not find any significant change in the BDNF. It seems that walking cannot increase the BDNF in patients with MCI, although the walking was intense. Among the studies that showed increases in the BDNF by the aerobic training, the protocols were as follows: intervention duration: 12 weeks, sessions per week: 3, intensity of training: 40% of HRmax, and time of training: 8 min.

The magnitude of the BDNF change in response to exercise depends on the blood lactate concentration and the duration and intensity of exercise and is transient in nature [15]. With exercise intensity considered important in determining the magnitude of change in physiological mechanisms, an intensity-driven change across these physiological mechanisms may be important for predicting the cognitive effect following acute exercise. Thus, if BDNF is a mediator of the effects of acute exercise on cognitive performance, intensity would be expected to influence behavioral outcomes. While initial results are promising that both maximal and submaximal intensities show positive effects on cognitive performance, further research is necessary to clarify the intensity effect.

The relationship between brain structure and cognitive ability can be elucidated from different perspectives, depending on the research question one is interested in. How the human brain works is still an open question, as is its implication with brain architecture: the non-trivial structure–function relationship [51]. The human brain has a large degree of plasticity, the capacity to adapt to changing demands by altering its structure [52]. Bidirectional dynamic interactions between the brain and behavior at different time scales are at the heart of cognitive life-span development [53].

Among researchers, the hippocampus is one of the most important structures in the brain to be considered. The results of studies on the volume of the hippocampus that were performed in neuropsychiatric disorders have many contradictions. MCI has been reported to be associated with hippocampal atrophy and memory loss [54,55]. Meanwhile, memory impairment is one of the most common symptoms in patients in the early stages of MCI [56]. Because exercise has been effective at reducing cortical decay in the elderly, Erickson et al. [57] looked at the relationship between exercise and hippocampal volume. Using MRI, they found that in 165 no-dementia older adults, there was a triple association of higher fitness levels, larger hippocampal volume, and better spatial memory performance for active individuals. The effect of exercise on the structure of the brain has various mechanisms that will be briefly discussed. Blood flow has been shown to increase during aerobic exercise in the body. In some regions of the cerebral cortex, blood flow and oxygen delivery (e.g., activation) are affected by aerobic exercise [58]. For example, activation in the prefrontal cortex (PFC), measured by brain oxygenation, increased during submaximal aerobic exercise (up to 80% of peak ability) [59]. In summary, evidence suggests that cerebral blood flow increases during low- to moderate-intensity exercise, leading to post-exercise changes in PFC activity and to improvements in various cognitive domains (e.g., executive function and processing speed) [59]. Aerobic exercise can also increase the immediate induction of markers of brain flexibility. Exercise-induced flexibility is associated with improved cognitive function, such as processing speed [60]. The plasticity mechanism may be through the exercise-induced release of neurotrophins [60]. BDNF is emerging as a key mediator for synaptic flexibility in the central computing center for memory processing (e.g., the hippocampus) and is thought to be modulated by the growth factor insulin-1 (IGF-1) [61]. DNF is thought to regulate synaptic proteins (such as synapsin I and synaptophysin) in the hippocampus, thereby improving axonal branching and increasing the effectiveness of synaptic transmission [62]. In humans, serum BDNF is commonly measured as an indirect indicator of neurogenesis. This is based on the evidence that BDNF produced in the brain accounts for 70 to 80% of circulating BDNF in response to aerobic exercise in humans [63]. In summary, motor flexibility in response to acute aerobic exercise may release BDNF neurotrophins, which are associated with improved cognitive function [63]. Preclinical data have also shown that aerobic exercise can affect the structure and function of the brain by increasing myokines [64]. Therefore, aerobic exercise through the mentioned mechanisms can be effective in improving MCI and help to improve it.

Among the reviewed studies (Table 3), two studies investigated the effect of aerobic training on hippocampal volume in patients with MCI. Two studies with high quality reported significant increases for hippocampal volume. Brinke et al. [29], for six months (training protocol; sessions per week: 3, intensity of training: 40 to 80% of HRmax of overload, time of training: 60 min) and Tarumi et al., for one year (training protocol; sessions per week: 3, intensity of training: 75 to 85% of HRmax, time of training: 25 to 30 min), investigated the effects of running on the hippocampal volume of patients with MCI, and both studies reported significant increases in hippocampal volume. Therefore, running with moderate intensity leads to a significant increase in hippocampal volume in adaptation. Earlier, Firth et al. [65], in a meta-analysis, confirmed the significant effects of aerobic exercise on hippocampal volume and stated that aerobic training interventions might be useful for preventing age-related hippocampal deterioration and maintaining neuronal health.

The aging brain is able to counterbalance structural attenuations by altering the functional recruiting patterns, thereby maintaining cognitive functions [66]. Such processes reflect the functional brain and cognitive plasticity in the aging human brain. Population-based studies have confirmed that individuals that stay physically active have an improved brain–behavior relationship [67]. Therefore, in patients with AD and MCI [68] and in older human subjects suffering mild memory loss [69], it has been shown that aerobic exercise can change electroencephalography (EEG) patterns and cerebral blood flow (CBF). Hence, aerobic exercise is recommended in patients with MCI [70].

Among the reviewed studies (Table 4), five studies investigated the effect of aerobic training on brain activity in patients with MCI. One study with high quality [13] (training protocol; type: aerobic dance, intervention duration: 3 months, sessions per week: 3, intensity of training: 60–80% of HRmax, time of training: 35 min), reported a non-significant change for the event-related potential. On the other hand, QI et al. [30] (high-quality study), with exactly the same training protocol, showed a significant increase in the amplitude of the low-frequency fluctuation in the bilateral fronto-temporal, entorhinal, anterior cingulate, and Para hippocampal cortex. In the other three studies, the results were inconsistent, meaning that some variables of brain activation improved significantly, while some were unchanged. Xia et al. [31] (high-quality study) investigated the effect of baduanjin exercise (training protocol; intervention duration: 24 weeks, sessions per week: 3, intensity of training: 55 to 75% of HRR max, time of training: 60 min) on the dorsal attention network in patients with MCI. The patients showed significant increases in the inferior parietal, precuneus, and fusiform gyrus, while non-significant changes were observed in the Rolandic operculum and the middle temporal gyrus. In another study, Thomas et al. [33] (high-quality study) investigated the changes in CBF caused by the effect of running (training protocol; intervention duration: 12 months, sessions per week: 3 to 5 of overload, intensity of training: 75 to 85% of HRmax, time of training: 25 to 40 min) in patients with MCI and showed a significant increase in the hippocampus and a significant decrease in the PCC, while non-significant changes were observed in the anterior cingulate cortex, frontal lobe, parietal lobe, temporal lobe, and occipital lobe. Moreover, Amjad et al. [32] investigated the brain waves in patients with MCI. After six weeks of running (training protocol; sessions per week: 3 to 5 of overload, intensity of training: 60 to 80% of HRmax, time of training: 20 to 40 min), a significant increase in alpha 2 waves and significant decreases in delta and beta 1 waves were observed, while non-significant changes were observed in theta, alpha 1, and beta 2 waves. Therefore, with these contradictory results and few studies, it is not possible to draw conclusions about the brain activity caused by aerobic exercise. 

Therefore, it can be said that one of the limitations of this study is that the results may not be generalizable to all patients with MCI because the studies selected only a few samples from the community of patients with MCI. Moreover, when reviewing their materials and methods, most of the studies did not state how they estimated their sample size. Therefore, studies may suffer from type II statistical errors (incorrect rejection of a true null hypothesis). In addition, some studies used only females [29,32] or males [27] as participants, making generalizing the results to all patients with MCI difficult. Meanwhile, another limitation of the reviewed studies is the heterogeneity of the MCI population. Moreover, some studies focused on Peterson’s MCI diagnostic criteria patients, but some studies used the neuropsychological test battery and clinical criteria, which maybe have major differences in the pattern of cognitive response.

In addition, based on the investigation effect of exercise training on a wide range of brain activity, a lack of consistent reporting, a heterogeneity of experimental design, and the varied types of exercise programs used in the reviewed studies, performing a meta-analysis was not possible. Moreover, the methods used to measure BDNF can be considered as a major limitation of some existing studies, which can lead to the heterogeneity of results, as BDNF can be monitored using a variety of tools and methods and in different sites. A further limitation is that only articles published in five databases (Web of science, PubMed, Cochrane Library, SCOPUS, and PEDro) in English peer-reviewed journals were included in the current study. Therefore, this review may not have been comprehensive enough to include all studies on the effects of aerobic training on brain plasticity in patients with MCI. However, the databases used in the current review study are major electronic databases that index a great number of studies. The majority of studies have neglected important notes in their methodology. For instance, nutritional considerations and the accurate assessment of the body composition were not considered complete. All these factors could influence outcomes and could help in the interpretation of the effects of aerobic training [48]. No studies reported any harmful effects of exercise in relation to brain-derived neurotrophic factor or structures, and aerobic training did not lead to inflammation or increase disease severity. Therefore, these findings indicate that an active lifestyle can be considered as an important part of MCI treatment.

The reviewed studies often used only a single type of exercise as an intervention, and there is no comparison between different types of exercise and intensities. Therefore, it is suggested that comparisons between different exercises should be made to determine the most effective exercise modality in terms of the effect on brain plasticity. Furthermore, there are significant requirements for high-quality studies that investigate both the short- and long-term effects of an active lifestyle on clinical and paraclinical parameters in patients with MCI. Although most studies did not show chronic effects of exercise on neural factors, it is suggested that the positive effects of exercise may be seen as the sum of the acute and chronic effects, leading to long-term improvements [71,72].

However, studies that were conducted on healthy subjects or even other diseases suggest a positive and modulating effect of exercise training on neural factors. Finally, there is an obvious gap in our understanding of the additive or synergistic roles of pharmaceutical therapies and exercise that are important for guiding future research. Overall, the limited and contradictory results that are summarized in the current study suggest that more extensive research is needed to better understand and quantify the role of aerobic training on brain plasticity in patients with MCI. However, this uncertainty is specifically about the effect of moderate- to high-intensity aerobic exercise in the long term on BDNF and brain structures in patients with MCI.

In addition, comparing aerobic exercise to other exercises such as resistance training can be very effective.

Based on the reviewed studies, it can probably be said that aerobic exercise is a cost-effective exercise that is associated with several physical benefits. The results of this study show that exercise also has a cognitive advantage for some adults with MCI. The enhancing effects of aerobic exercise on MCI can be enhanced when exercise is moderate and more intense than running. It also seems that the duration of training between 20 to 30 minutes on average in each training session, can be effective. We also think that older people should be aware of the duration of aerobic exercise. However, more research on aerobic exercise is necessary.

## Figures and Tables

**Table 1:** Characteristics and protocols of the reviewed studies.

		Brain Neurotrophic									
Authors	Year	Participants				Type of Aerobic Exercise	Intervention Duration	Sessions Per Week	Intensity of Training	Time of Training	Control Group
		Sample	Age (Mean ± SD)	MMSE	MoCA						
Fungwe et al. [12]	(2019)	Male: (*n* = 12) Female: (*n* = 5)	71.19 ± 6.9	25.74 ± 2.0		Endurance training	6 months	3	50% to 70% VO_2max_ of OL	20 to 40 min of OL	Control group with supervised stretch exercise
Damirchi et al. [11]	(2018)	Female: (*n* = 24)	68.96 ± 4.3	23.31 ± 2.1		Walking	8 weeks	3	55% to 75% HRmax of OL	6 min to 20 min of OL	Usual care only
Kohanpour et al. [27]	(2017)	Male: (*n* = 20)	67.85 ± 3.8			Running	12 weeks	3	75 to 85% HRmax	8 to 26 min of OL	Usual care only
Allard et al. [26]	(2016)	Male: (*n* = 7) Female: (*n* = 15)	72.00 ± 7.2			Running	6 months	3	50% to 70% VO_2max_ of OL	20 to 40 min of OL	Stretch training
Baker et al. [17]	(2010)	Male: (*n* = 14) Female: (*n* = 15)	70.35 ± 7.5	27.45 ± 1.9		Running	6 months	4	40% to 80% HRmax of OL	45 to 60 min of OL	Stretching activities
Tarumi et al. [28]	(2019)	Male: (*n* = 27) Female: (*n* = 43)	64.65 ± 6.2	28.95 ± 1.2		Running	12 months	3	75 to 85% HRmax	25–30 min	Stretching and toning program
Brinke et al. [29]	(2014)	Female: (*n* = 39)	64.65 ± 6.2	28.95 ± 1.2		Running	12 months	3	75 to 85% HRmax	25–30 min	Stretching and toning program
Thomas et al. [33]	(2020)	Male: (*n* = 16) Female: (*n* = 14)	66.25 ± 6.9	29.25 ± 0.9		Running	12 months	3 to 5 of OL	75 to 85% HRmax	25 to 40 min	Stretch training
Xia et al. [31]	(2019)	Male: (*n* = 12) Female: (*n* = 34)	65.82 ± 4.8		21.56 ± 2.8	Baduanjin exercise	24 weeks	3	55 to 75% HRmax	60 min	Usual physicalactivity control group
Zhu et al. [13]	(2018)	Male: (*n* = 24) Female: (*n* = 36)	69.65 ± 7.0		23.05 ± 2.0	Aerobic dance	3 months	3	60–80% of HRmax	35 min	Usual care only
QI et al. [30]	(2018)	Male: (*n* = 17) Female: (*n* = 33)	69.85 ± 7.1	27.20 ± 1.2	22.75 ± 1.9	Aerobic dance	3 months	3	60–80% of the HRmax	35 min	Usual care only
Amjad et al. [32]	(2018)	Male: (*n* = 21) Female: (*n* = 19)	58.89 ± 2.4	24.17 ± 0.8	20.94 ± 0.7	Running	6 weeks	3	60 to 80% HRmax	20 to 40 min of OL	Usual care only

MMSE: Mini–Mental State Examination, MoCA: Montreal Cognitive Assessment, OL: Overload, HHR: Heart Rate Reserve.

**Table 2:** The overall results of BDNF in reviewed studies.

Authors		Number of Participants		Unit	Intragroup Comparison		Results
	Year	Experimental Group	Control Group		Pre-Test	Post-Test	
Fungwe et al. [2]	(2019)	10	7				↑
Allard et al. [8]	(2016)	13	9	ng/mL	76.3 ± 28.3		↑
Damirchi et al. [3]	(2018)	11	9	pg/mL	1167.46 ± 473.91	1122.41 ± 542.66	↔
Baker et al. [7]	(2010)	19	10	pg/mL			↑
Kohanpour et al. [12]	(2017)	10	10	pg/mL	110.25 ± 28.61	192.84 ± 59.51	↑

↑: Significant increase, Unchanged: ↔.

**Table 3:** The overall results of the brain structures in reviewed studies.

Authors	Year	Results
Brinke et al. [29]	(2014)	Hippocampus Volume ↑
Tarumi et al. [28]	(2019)	Hippocampus Volume ↑

↑: significant increase.

**Table 4:** The overall results of the brain activity in reviewed studies.

Authors	Year	Outcomes and Their Measures	Results
Thomas et al. [33]	(2020)	Event-related potential: EEG	Event-related potential ↔
Xia et al. [31]	(2019)	ALFF in the bilateral fronto-temporal, entorhinal, anterior cingulate, and parahippocampal cortex: fMRI	ALFF in the bilateral fronto-temporal ↑, entorhinal ↑, anterior cingulate ↑, parahippocampal cortex ↑
Zhu et al. [13]	(2018)	Cerebral blood flow: MRI	ACC ↔, PCC ↓, Hippocampus ↑, Frontal lobe ↔, Parietal lobe ↔, Temporal lobe ↔, and Occipital lobe ↔
QI et al. [30]	(2018)	Dorsal attention network: MRI	IPL ↑, ROL ↔, MTG ↔, PCUN ↑, and FFG ↑
Amjad et al. [32]	(2018)	Waves: EEG	Delta ↓, Theta ↔, Alpha 1 ↔, Alpha 2 ↑, Beta 1 ↓, and Beta 2 ↔

EEG: Electroencephalography, ALFF: Amplitude of low-frequency fluctuation, fMRI: Functional Magnetic Resonance Imaging, MRI: Magnetic Resonance Imaging, ACC: Anterior Cingulate Cortex, PCC: Posterior Cingulate Cortex, IPL: Inferior Parietal, ROL: Rolandic Operculum, MTG: Middle Temporal Gyrus, PCUN: Precuneus, FFG: Fusiform Gyrus, ↑: significant increase, ↓: significant decrease, Unchanged: ↔.

**Table 5:** The methodological quality of the reviewed studies using the PEDro scale.

Scales												
Authors	#1	#2	#3	#4	#5	#6	#7	#8	#9	#10	#11	Total
Zhu et al. [13]	1	1	1	1	0	0	1	1	0	1	1	7
Fungwe et al. [12]	1	1	0	1	0	0	1	0	0	1	1	5
Damirchi et al. [11]	1	1	0	1	0	0	0	0	1	1	1	5
QI et al. [30]	1	1	0	1	0	0	0	0	1	1	1	5
Xia et al. [31]	1	1	0	1	0	0	1	1	1	1	1	7
Brinke et al. [29]	1	1	1	1	0	0	1	0	1	1	1	7
Baker et al. [17]	1	1	0	1	0	0	1	1	0	1	1	6
Allard et al. [26]	1	1	0	1	0	0	1	1	1	1	1	7
Tarumi et al. [28]	1	1	0	1	0	0	1	0	1	1	1	6
Thomas et al. [33]	1	1	0	1	0	0	1	1	1	1	1	7
Amjad et al. [32]	1	1	0	1	0	0	1	0	0	1	1	5
Kohanpour et al. [27]	1	1	0	1	0	0	0	0	0	1	1	4

#1: Eligibility criteria (not included in the total score); #2: Random allocation; #3: Allocation was concealed; #4: The groups were similar in important criteria at the baseline; #5: Blind all subjects; #6: Blind therapy administration; #7: Blind all assessors; #8: Outcomes were obtained from more than 85%; #9: Intention to treat analysis; #10: Statistical comparisons between groups for at least one key factor; #11: Point estimates and variability.

## Data Availability

Data sharing is not applicable to this article as no new data were created or analyzed in this study.
